# Enhancing Lipidomics With High-Resolution Ion Mobility-Mass Spectrometry

**DOI:** 10.1002/pmic.70026

**Published:** 2025-08-13

**Authors:** Gaoyuan Lu, Shuling Xu, Penghsuan Huang, Lingjun Li

**Affiliations:** 1 School of Pharmacy, University of Wisconsin-Madison, Madison, Wisconsin, USA; 2 Department of Chemistry, University of Wisconsin-Madison, Madison, Wisconsin, USA; 3 Lachman Institute for Pharmaceutical Development, School of Pharmacy, University of Wisconsin-Madison, Madison, Wisconsin, USA; 4 Wisconsin Center for NanoBioSystems, School of Pharmacy, University of Wisconsin-Madison, Madison, Wisconsin, USA

## Abstract

Lipids, indispensable yet structurally intricate biomolecules, serve as critical regulators of cellular function and disease progression. Conventional lipidomics, constrained by limited resolution for isomeric and low-abundance species, has been transformed by ion mobility-mass spectrometry (IM-MS). This technology augments analytical power through enhanced orthogonal separation, collision cross-section (CCS)-based identification, and improved sensitivity. This review examines the transformative advances in IM-MS-driven lipidomics, focusing on three major pillars: (1) a critical evaluation of leading ion mobility spectrometry (IMS) platforms, emphasizing innovative instrument geometries and breakthroughs in resolving lipid isomers; (2) an exploration of lipid CCS databases and predictive frameworks, spotlighting computational modeling and machine learning strategies that synergize experimental data with molecular representations for high-confidence lipid annotation; (3) emerging multi-dimensional lipidomics workflows integrating CCS with liquid chromatography-MS/MS to boost identification and depth, alongside mass spectrometry imaging for spatially resolved lipidomics. By unifying cutting-edge instrumentation, computational advances, and biological insights, this review outlines a roadmap for leveraging IM-MS to unravel lipidome complexity, catalyzing biomarker discovery and precision medicine innovation.

## 1 | Introduction

Lipids, a diverse and dynamic class of biomolecules, are indispensable to life. They orchestrate cellular energy storage, membrane architecture, and signaling cascades while serving as biomarkers for metabolic diseases, cancer, and neurodegenerative disorders [1, 2]. However, the structural complexity of lipids—marked by variations in acyl chain length, chain connectivity, double bond numbers, position, and stereochemistry—poses a formidable analytical challenge [3, 4]. Traditional lipidomics workflows, relying on mass spectrometry (MS) coupled with chromatography, often struggle to resolve isomeric species, detect low-abundance lipids, or map their tissue-specific localization [5]. These limitations underscore the urgent need for technologies that enhance separation power, identification confidence, and spatial resolution in lipid analysis.

Ion mobility spectrometry (IMS), an advanced gas-phase separation technique that resolves ions based on their size, shape, and charge [6–8], provides the following three distinct advantages compared to conventional mass spectrometry methods for lipid analysis. First, this separation technique introduces an orthogonal separation dimension that differentiates isobaric and isomeric lipids, which often co-elute in traditional chromatographic or MS-only workflows. Second, IMS generates collisional cross-section (CCS) values—a reproducible physicochemical parameter that reflects an ion’s gas-phase mobility and represents its conformational structures, which serves as a robust metric for lipid species identification. Third, IMS enhances sensitivity by improving both signal-to-noise ratios and peak capacity, which significantly increases the ability to filter noise and detect low-abundance lipid species. Over the past decade, advancements in IMS instrumentation have revolutionized lipidomics by achieving unprecedented resolution (greater than 200) and attomole-level detection sensitivity [8]. Additionally, the development of comprehensive CCS databases, machine learning models, and multidimensional identification frameworks has elevated lipid annotation from tentative assignments to detailed, structurally resolved characterizations.

While prior reviews have highlighted IMS applications in lipidomics [5, 9–12], this review uniquely synthesizes three interconnected pillars of recent progress in lipid analysis. In this review, we begin by evaluating leading IMS platforms—including the Agilent 6560 drift tube IMS, Waters cyclic IMS, Bruker timsTOF, and several other emerging systems—emphasizing their unique capabilities in isomer resolution. This comparative assessment illustrates the performance of these advanced instruments, guiding readers in selecting the most suitable IMS tool for their experimental objectives. The second section delves into the accurate measurement of CCS values for lipids and discusses methods for generating or predicting CCS values for lipid species that lack fully characterized experimental structures. The integration of CCS measurements with expanding CCS libraries has significantly broadened lipidomics coverage, enabling more precise identification and deeper insights into lipid metabolism. Finally, we review recent advances in lipidomics through both chromatography-based four-dimensional (4D) approaches (incorporating retention time [RT], MS1, MS/MS, and CCS) and mass spectrometry imaging-based spatial lipidomics. By integrating technological, computational, and omics advancements, this review provides a comprehensive roadmap for harnessing the power of IMS to reveal the full complexity of the lipidome.

## 2 | High-Resolution IMS Platforms for Lipid Analysis

### 2.1 | Principle of IMS

IMS separates gas-phase ions based on their size, shape, and charge prior to mass analysis [6–8]. This separation occurs in a cell filled with neutral buffer gas, where an electric field propels the ions. An ion’s transit time is governed by its ion mobility coefficient (K), which is inversely related to its rotationally averaged collision cross-section (CCS, Ω). Consequently, compact ions with smaller CCS values migrate faster than larger, more extended ions, enabling their separation. There are three high-resolution IMS platforms commonly used in lipid analysis: drift tube IMS (DTIMS), traveling wave IMS (TWIMS), and trapped IMS (TIMS).

**FIGURE 1 |:**
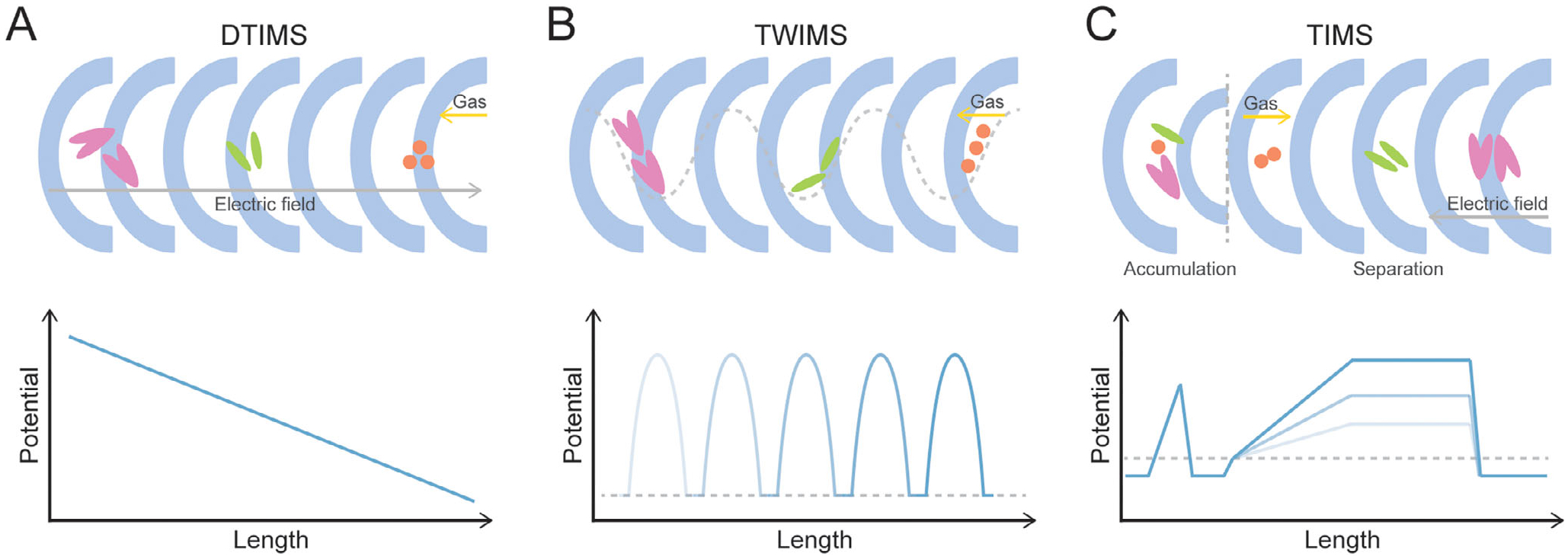
Schematic overview of three common ion mobility spectrometry (IMS) techniques used in lipid analysis. (A) Drift Tube IMS (DTIMS) utilizes a uniform electrostatic field to separate ions against a constant flow of buffer gas. (B) Traveling Wave IMS (TWIMS) uses propagating voltage waves to push ions through the cell, separating them based on their mobility. (C) Trapped IMS (TIMS) first accumulates ions using a gas flow directed against an electric field barrier, then separates the ions by sequentially releasing them as the barrier potential is lowered.

DTIMS is the foundational ion mobility technique (Figure 1A). It employs a uniform, static electric field within a drift tube of known length. Ions achieve a constant velocity when the electric force is balanced by frictional drag from the buffer gas. The measured drift time is directly proportional to the ion’s CCS. TWIMS utilizes a series of propagating voltage waves to propel ions through a gas-filled cell (Figure 1B). The dynamic electric field pushes ions along, and their ability to keep pace with the waves depends on their mobility. Higher mobility ions travel faster through the device than lower mobility ions, resulting in separation. TIMS operates by trapping ions against a flow of buffer gas using an opposing, non-uniform electric field (Figure 1C). An ion is held stationary where the drag force from the gas flow is perfectly balanced by the force from the electric field, a position dictated by the ion’s specific mobility. Separation is achieved by gradually reducing the electric field strength, which sequentially releases the trapped ions in order of increasing CCS (from highest to lowest mobility).

In the following section, we will provide a detailed overview of the most advanced mass spectrometry platforms available for the three types of IMS, as well as for other IMS technologies.

### 2.2 | Agilent 6560 Drift Tube IMS

DTIMS, recognized as the pioneering ion mobility technique, operates by applying a uniform electric field across a drift tube filled with neutral buffer gas. This method is grounded in the classical Mason-Schamp equation [13], which establishes a direct relationship between an ion’s drift time and its CCS. By leveraging this theoretical framework, DTIMS uniquely enables precise CCS determination ab initio (from first principles) without dependence on external calibration standards [7]. Specifically, CCS values are derived from fundamental physical parameters such as drift time, electric field strength, gas pressure, and temperature, allowing direct calculation of reduced mobility (*K*_0_) [13]. This intrinsic independence from calibrant ions positions DTIMS as the gold standard for CCS measurement, offering unparalleled accuracy and reproducibility, as discussed in a later section. A prominent commercial implementation of DTIMS, the Agilent 6560 Ion Mobility Quadrupole Time-of-Flight (Q-TOF) system, has significantly advanced structural characterization and omics research by delivering high-accuracy CCS data [14].

The Agilent 6560 ion mobility platform operates in two distinct modes: single-pulse mode and demultiplexed mode, each offering unique analytical trade-offs. In single-pulse mode, ions are introduced as a single packet into the drift tube, achieving a resolution of approximately 50. While this mode enables differentiation of ions based on drift time differences, it often fails to achieve baseline separation for structurally similar lipid isomers (e.g., those differing in double bond or acyl chain positions). To mitigate this limitation, strategic adduction with metal ions (e.g., sodium) can be employed to amplify drift time disparities between isomers [15], thereby improving their distinguishability.

**FIGURE 2 |:**
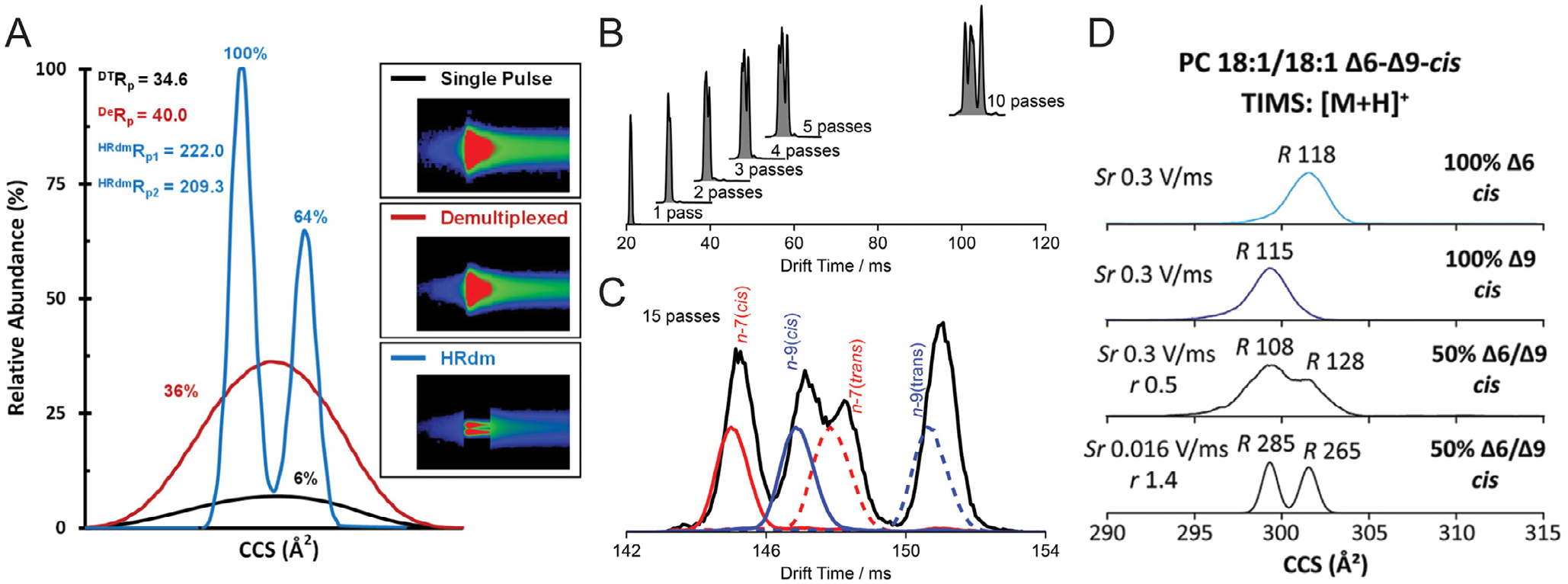
(A) Performance evaluation of the Agilent 6560 drift tube IMS platform using PC 38:6. Demultiplexed acquisition combined with HRdm algorithmic deconvolution demonstrates enhanced ion mobility resolution and signal intensity relative to conventional single-pulse mode acquisition. Adapted from Koomen et al. [17] with permission. (B, C) Separation of four fatty acid (FA) isomers—FA 18:1*n*-7 (*cis*), FA 18:1*n*-7 (*trans*), FA 18:1*n*-9 (*cis*), and FA 18:1*n*-9 (*trans*)—on the Waters cyclic IMS platform. Progressive increases in the number of passes (1 to 15) improve isomer resolution. Adapted from Poad et al. [19] with permission. (D) Resolving double bond positional isomers of PC 18:1/18:1 using the Bruker timsTOF platform. Reduced scan rates (Sr) correlate with increased resolution. Adapted from Jeanne Dit Fouque et al. [23] with permission.

In contrast, demultiplexed mode enhances analytical performance by injecting multiple ion packets into the drift tube at predefined intervals. The overlapping ion signals generated in this approach are computationally resolved using Hadamard Transformation, a mathematical deconvolution algorithm that reconstructs high-resolution demultiplexed (HRdm) data [16]. This process significantly improves both sensitivity and resolution: for example, Koomen et al. [17] reported a six-fold increase in sensitivity and a significant enhancement in resolution (from 35 in single-pulse mode to 210 in HRdm mode) for the lipid species PC 38:6 when applying 4-bit multiplexing with HRdm algorithmic processing (Figure 2A). The improved limit of detection and resolution in demultiplexed mode make it particularly advantageous for analyzing low-abundance isomers or complex biological mixtures.

### 2.3 | Waters Cyclic IMS

Cyclic Ion Mobility Spectrometry (CIMS) revolutionizes analytical resolution by enabling ions to traverse multiple cycles within a circular TWIMS cell, effectively extending the ion mobility path length. This design allows the resolution to be dynamically tuned by adjusting the number of cycles, ranging from ~60 (for a single pass) to over 750 after 100 passes, making CIMS uniquely suited for targeted ultrahigh-resolution separations [18]. However, the non-uniform electric field inherent to the circular TWIMS cell necessitates careful calibration to ensure accurate CCS determination. While cumulative ion losses (~2.5% per pass) impose practical limits on cycle counts, the technique demands analyte stability to mitigate sensitivity losses during extended separations [18].

CIMS has emerged as a transformative tool for isomer-resolved lipidomics. For example, Poad et al. [19] employed multi-pass CIMS to resolve lipid isomers in prostate cancer cell extracts, distinguishing subtle structural variations such as carbon-carbon double bond position, geometry (*cis/trans*), and acyl chain *sn*-position. In a mixture of four fatty acid (FA) isomers—FA 18:1*n*-7(*cis*), FA 18:1*n*-7(*trans*), FA 18:1*n*-9(*cis*), and FA 18:1*n-*9(*trans*)—single-pass CIMS yielded a single merged mobility peak. However, increasing the separation to 15 cycles, combined with esterification-based derivatization, resolved all four isomers at a resolution of ~150 (Figure 2B,C). Similarly, de Las Heras Prieto et al. [20] achieved baseline separation of mycolic acid isomers using up to 70 passes, underscoring CIMS’s capability to address previously intractable isomer complexity.

Beyond multi-pass separations, CIMS enables mobility-selective isolation and tandem IMS (IMSn) workflows. In IM isolation mode, ions outside a predefined mobility range are ejected, while selected ions undergo additional cycles for enhanced resolution. For instance, de Bruin et al. [21] demonstrated the use of CIMS to resolve triacylglycerol (TG) isomers. Following an initial 20-pass separation, a mixture of four TG isomers produced two partially resolved peaks: TG(16:0/18:1(9Z)/16:0) / TG(16:0/18:1(9E)/16:0) and TG(16:0/16:0/18:1(9Z)) / TG(16:0/16:0/18:1(9E)). By isolating these peaks and subjecting them to a second round of separation (60 passes), baseline resolution of all four isomers was achieved. This hierarchical approach highlights the scalability and adaptability of CIMS for analyzing complex mixtures.

Despite its advantages, CIMS faces inherent trade-offs. Extended ion transit times reduce transmission efficiency, particularly at high cycle counts (>50), while spatial compression of ion packets during cycling (the “wrap-up” effect) can distort mobility measurements [18]. Nevertheless, CIMS’s adjustable resolution and compatibility with advanced workflows position it as a cornerstone technology for advancing structural lipidomics and omics sciences.

### 2.4 | Bruker timsTOF

Unlike traditional ion mobility spectrometry, where ions travel through an inert gas, TIMS immobilizes ions within a gas flow inside a segmented quadrupole ion trap [22]. Separation is achieved by dynamically modulating electric field gradients across parallel plates, with ions of larger CCS being displaced first by the gas flow. Subsequent elution—controlled by precise adjustments to the plate potentials—enables either high-resolution separations or rapid scanning modes. When properly calibrated, TIMS provides CCS values with ~1% relative accuracy, rivaling the precision of DTIMS. Furthermore, an optimized step-scan function can achieve an IMS resolution exceeding 300 while reducing experimental time and improving the duty cycle [8].

For example, Jeanne Dit Fouque et al. [23] demonstrated TIMS for the separation of PC 18:1/18:1 Δ6-*cis* and PC 18:1/18:1 Δ9-*cis* isomer mixtures (Figure 2D). At a standard scan rate of 0.3 V/ms, the ion mobility resolution was approximately 110—insufficient to resolve the double-bond positional isomers. However, by fine-tuning the scan rate to 0.016 V/ms, the resolution increased to 280, enabling near-baseline separation of the two isomers.

The integration of Parallel Accumulation-Serial Fragmentation (PASEF) in Bruker’s timsTOF platform further enhances analytical performance by optimizing the duty cycle, ensuring unmatched MS/MS coverage. Remarkably, TIMS can achieve a 100% duty cycle in optimized configurations [24], a critical advantage for the sensitivity and high-throughput demands of lipidomics. For instance, Vasilopoulou et al. [25] demonstrated the potential of PASEF technology to increase lipidome coverage and lower detection limits. However, the accurate assignment of lipid features depends on strict quality control, which will be discussed in a later section.

Zhu and colleagues [26] systematically evaluated the performance of lipid identification with and without TIMS separation using NIST human plasma samples. Their study demonstrated that TIMS-based multidimensional separation significantly enhanced the differentiation of isomeric and isobaric lipids. Additionally, it improved the purity of precursor ion isolation and the quality of MS/MS spectra. For instance, without TIMS, the precursors of LPC (O-16:1) and LPE (18:1) were co-isolated, resulting in a chimeric MS/MS spectrum. In contrast, TIMS enabled its sequential elution based on distinct 1/K_0_ values. In positive ion mode, a total of 32,925 MS/MS spectra were acquired using LC-MS without TIMS, whereas LC-TIMS-MS generated 67,553 spectra from the same plasma samples. Consequently, 366 lipid species were identified without TIMS, compared to 729 species identified with TIMS separation. These identifications were refined using fragmentation rule-based approaches to ensure accurate structural elucidation.

Beyond its inherent compatibility with (nano)electrospray ionization (ESI), recent advancements have enabled Bruker timsTOF to seamlessly switch between ESI and matrix-assisted laser desorption/ionization (MALDI), significantly broadening its utility in spatial lipidomics research, as demonstrated in a later section.

### 2.5 | Other IMS Types

Structures for Lossless Ion Manipulation (SLIM) represent an emerging advancement in ion mobility separation, leveraging traveling wave technology within an extended ~13-m serpentine path [27]. This design capitalizes on the fundamental principle that resolution scales with the square root of the separation path length, enabling SLIM to achieve exceptional resolution for complex lipid isomers. A landmark study by May et al. [28] demonstrated SLIM’s analytical potential by resolving isomeric triacylglycerols and gangliosides with an ion mobility resolution of approximately 300. Subsequent work by Rose et al. [29] established an SLIM-based CCS calibration framework tailored for lipids, while Reardon et al. [30] applied SLIM to intricate lipid mixtures, achieving baseline separation of geometric *cis/trans* isomers, double-bond positional isomers, and *sn*-positional stereoisomers. Further expanding its utility, Wormwood Moser et al. [31] employed SLIM to explore the structural diversity of ganglioside lipidomes, underscoring its versatility in lipidomics.

Differential Mobility Spectrometry (DMS), or Field Asymmetric Waveform Ion Mobility Spectrometry (FAIMS), differentiates ions based on the nonlinear dependence of their mobility on electric field strength [32]. By applying high-frequency asymmetric waveforms, DMS selectively transmits ions according to their differential mobility coefficients under alternating high- and low-field conditions [32]. Bowman et al. [33] advanced lipid analysis using DMS by systematically optimizing parameters such as drift gas composition and flow rate, achieving baseline separation of lipid classes including diacylglycerols (DG), phosphatidylcholines (PC), and triacylglycerols (TG). This work highlights DMS’s adaptability for targeted lipid separations under tailored experimental conditions.

The recently developed U-shaped Mobility Analyzer (UMA) introduces a novel geometric configuration to ion mobility technology. While retaining counter-flow principles common to differential mobility spectrometers, UMA features a bifurcated design with two parallel ion channels (CH1 and CH2) formed by segmented electrode arrays. Both channels maintain identical gas flow vectors (direction and velocity), guiding ions along a U-shaped trajectory that enhances separation efficiency [34]. Li et al. [35] demonstrated UMA’s analytical potential by integrating its separation capabilities with Aza-Prilezhaev aziridination derivatization chemistry. This hybrid approach resolved challenging isomeric lipid species that conventional IMS could not differentiate at comparable resolution levels, showcasing UMA’s promise for addressing complex lipidomic challenges.

### 2.6 | Challenges and Strategies for Lipid Analysis in IMS

When separating lipid isomers using IMS, the following equation can be used to estimate separation efficiency:

$$R_{pp}\left(\text{predicted}\right)=0.00589\times R_{p}\times \Delta CCS\%$$

In this equation, *R*_*p*_ represents the ion mobility resolution, and *R*_*pp*_ represents the predicted two-peak resolution between isomers. A value of 2.0 corresponds to complete baseline separation (100%), 1.23% to 90% separation, 0.93% to 50% separation, and values below 0.5 suggest little to no separation. This relationship underscores the fact that isomers with smaller differences in CCS require higher IMS resolution for effective separation. For example, McLean and colleagues [36] compiled a comprehensive CCS database of biomolecules based on the fundamental low-field ion mobility equation. Within their dataset, ~20 protonated lipid and lipid-like isomer groups in the [M + H]^+^ form show a wide range of CCS differences, from as little as 0.05% (e.g., between 21-hydroxypregnenolone and 17*α*-hydroxypregnenolone) to as much as 3.34% (e.g., between cortisone and aldosterone). Achieving 90% separation for the latter pair would require an IMS resolution of approximately 60—easily attainable on modern platforms. In contrast, resolving isomers with a 0.05% CCS difference would necessitate an IMS resolution exceeding 4000, which is well beyond current instrument capabilities.

Such minimal CCS differences present a significant challenge not only for separation but also for the reproducibility and accuracy of CCS measurements. Reports indicate that traditional stepped-field DTIMS methods exhibit a relative standard deviation (RSD) of ~0.29% across laboratories [37]. Therefore, when the CCS difference between two isomers falls below this threshold, reliable differentiation becomes difficult due to intrinsic measurement uncertainty.

Despite these limitations, alternative approaches have been explored to enhance isomer separation. Derivatization and metal ion adduction are commonly used to amplify structural differences [38, 39]. Derivatization chemically modifies lipids, potentially increasing CCS disparities. Similarly, adduction with specific metal ions can influence both the gas-phase conformation and fragmentation behavior of lipids, thus enhancing isomer discrimination. For example, Naylor et al. [39] developed a comprehensive toolkit combining permethylation and metal ion adduction, enabling full separation of isomeric sphingolipids and ceramides on a CIMS platform. However, metal addition does not always result in improved separation. In some cases, it may reduce CCS differences as seen with the [M + Na]^+^ form of PC 16:1/16:1 Δ9-*cis/trans*, which exhibits smaller CCS differences than their [M + H]^+^ counterparts. Hence, it is advisable to examine multiple ion adduct forms (e.g., [M + H]^+^, [M + Na]^+^, [M + K]^+^) and possibly switch ionization polarities during analysis.

An additional factor to consider is the potential formation of gas-phase conformers. A striking example is 25-hydroxyvitamin D3 (25OHD3), which in the sodium-adducted form exhibits two baseline-separated peaks in the ion mobilogram [40]. These peaks likely correspond to conformers with sodium ions bound at different sites. This behavior enables its rapid separation from its epimer, 3-epi-25OHD3, which exhibits only a single peak.

Historically, one of the major limitations of IMS–MS has been its sensitivity, primarily constrained by ion transmission losses and low duty cycles [41]. Ion mobility cells operate at relatively high pressures compared to the downstream mass analyzer, and efficiently transferring ions across this pressure gap is non-trivial. Advanced ion optics, including ion funnels and trapping devices, are critical to mitigating these losses [22]. Additionally, many traditional IMS instruments suffer from low duty cycles due to the pulsed nature of ion packet analysis. However, recent innovations have addressed this issue. For instance, in DTIMS, multiplexing techniques significantly improve duty cycles [17]. More impressively, TIMS combined with PASEF acquisition enables nearly 100% duty cycle, vastly enhancing sensitivity [24].

Regarding throughput, IMS is inherently fast, operating on a millisecond timescale. However, overall analysis time is typically dominated by slower front-end separations, such as liquid chromatography (LC). Because IMS can be nested within the LC elution window without extending total analysis time, improvements in throughput should primarily focus on optimizing these upstream separation strategies.

## 3 | Accurate Lipid CCS Measurement and Predictive Modeling

### 3.1 | Experimental CCS Measurements: Precision, Advances, and Challenges

**FIGURE 3 |:**
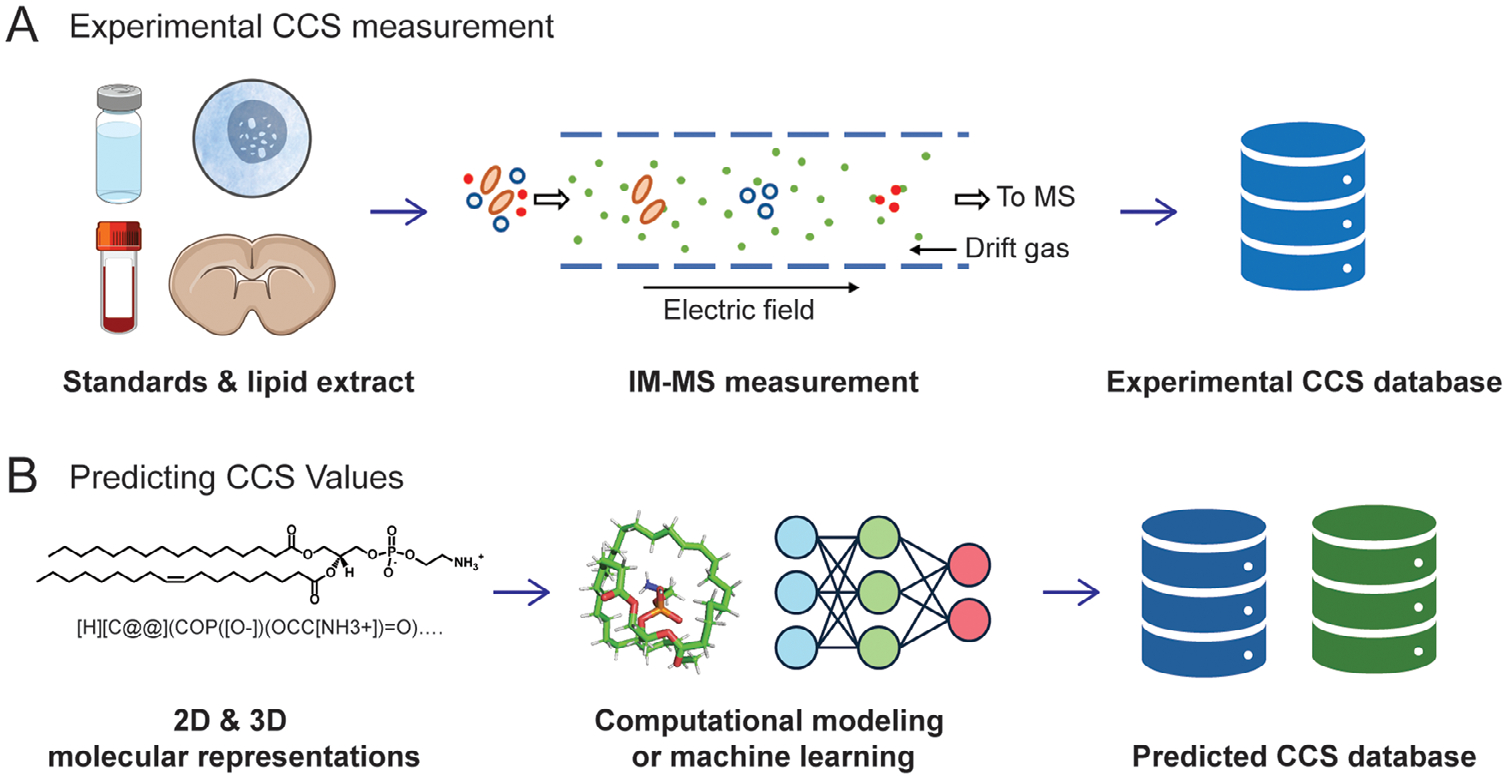
Two major strategies for the construction of CCS databases: (A) experimental measurement; (B) prediction via computational modeling or machine learning.

CCS values, derived from IM-MS, provide critical structural insights for lipidomics. Experimental CCS values, typically measured using DTIMS, TWIMS, or TIMS, are highly reproducible, with some methodologies achieving RSD below 0.3% [42, 43]. Over the past decades, the primary method for constructing CCS databases has been the experimental measurement of CCS values using chemical standards. However, commercial standards are often limited and expensive; many studies constructed the lipid CCS database using the lipid extracts from complex biological samples (Figure 3) [44]. Table 1 summarizes three experimental lipid CCS databases (Lipid MAPS, CCS base [45], METLIN-CCS [46]) constructed from previously published data, encompassing a broad range of measured lipid ion adduct forms.

In addition, experimental CCS measurements are constrained by instrument-specific calibration and inter-laboratory variability. To harmonize CCS values for confident identification, it is necessary to minimize the deviations of CCS between databases. IMS employs two primary approaches for measuring CCS values: calibrant-independent and calibrant-dependent methods. The stepped-field method in DTIMS is the only calibrant-independent approach, enabling direct CCS measurement based on the Mason–Schamp equation [7, 47]. This technique involves conducting IM experiments at multiple drift voltages to establish a linear relationship between measured drift times and the inverse drift voltages (1/*V*). The CCS value is then derived from the slope of the fitted linear regression curve. The precision of CCS measurements using this method depends on factors such as drift tube length, experimental temperature, and drift voltage accuracy. Consequently, the stepped-field method is regarded as the gold standard for CCS determination. Despite its accuracy, the stepped-field method is not compatible with chromatographic separation, limiting its applicability in liquid chromatography (LC)–IM–MS workflows. To overcome this issue, Kurulugama et al. [48] introduced a single-field method for DTIMS, which requires only one drift voltage for CCS determination. This approach first establishes a calibration curve by measuring reference compounds with known CCS values (e.g., Agilent ESI Low-concentration Tune Mix). Two key parameters, the mobility-independent flight time (*t*_fix_) and an instrument-dependent proportionality coefficient (*β*), are derived from this calibration. CCS values for analyte ions are subsequently calculated using their measured drift times. Recent studies have shown that the single-field method achieves an average error of 0.54% compared to the stepped-field method [37]. In addition, the inter-laboratory evaluation reported that the calibrated single-field CCS method provided an average error of 0.27% for lipids. Its compatibility with LC–IM–MS workflow has made it a widely adopted technique in lipidomics research. For example, Zhu and co-workers employed the single-field method in LC-IM-MS workflow for glycerophospholipids (GP), sphingolipids (SP), glycerolipids (GL), and sterol lipids (SL) CCS measurement [44, 49]. Baker and colleagues also utilized this method to measure the CCS values of five lipid categories (GP, SP, GL, SL, and fatty acids) in plasma and bronchoalveolar lavage fluid [50, 51]. This workflow was also applied by the Li group for GP CCS determination in the mouse brain [52, 53].

TIMS determines CCS values using a calibrant-dependent approach based on ion trapping and selective release. Unlike DTIMS, TIMS traps ions in a pressurized region using an electric field against a counter-flowing buffer gas. By gradually decreasing the electric field, ions are sequentially released in order of increasing mobility. The relationship between ion mobility and CCS is established through calibration with reference compounds of known CCS values. A calibration curve is generated by plotting the mobility of calibrants against their corresponding CCS values, allowing the derivation of a regression model. Using this model, CCS values of analyte ions are determined based on their measured mobilities. Herein, the accuracy of CCS measurements in TIMS is influenced by the choice of calibrants. Agilent ESI Low-concentration Tune Mix is also the most used calibrant in TIMS [25]. To validate the TIMS CCS values, Baker et al. [46] compared the TIMS CCS values against DTIMS CCS values. The results demonstrated an average CCS variation of ±1.03% for lipids, indicating good correlation and similar CCS values between the two IMS techniques for these molecule types [46]. While TIMS provides reliable CCS values, continued refinement of calibration strategies and expansion of experimental CCS databases will be essential for improving measurement precision and broadening its applicability in lipidomics. Similarly, TWIMS relies on a calibrant-dependent approach for CCS measurement. The accuracy of TWIMS-based CCS measurements is highly dependent on the structural similarity between analytes and calibrants. Hines et al. [54] revealed that using structurally mismatched calibrants can introduce large errors in lipid CCS measurements. For instance, lipid CCS values derived using calibrants including polyalanine (PolyAla), tetraalkylammonium salts (TAA), and hexakis(fluoroalkoxy)phosphazines (HFAP) exhibited a large relative error (up to 6.4%) compared to DTIMS-based CCS values, whereas lipid-based calibrants using phosphatidylethanolamines (PE) and PC standards reduced this error to below 2% [54]. Thus, the CCS values reported from different groups could have deviations and may be suitable for specific types of instruments.

Currently, many research groups are devoted to improving calibration approaches for standardization of the CCS determination [55]. Advances in calibrant selection, improved drift gas conditions, and enhanced data acquisition methods have addressed some of these limitations. In the future, standardizing the CCS measurement and expanding the quantity of empirical measurements by a joint effort from the lipidomics community would be an invaluable progress to support high-confidence lipid characterization.

### 3.2 | Predicting CCS Values: Integrating Computational Models and Machine Learning

Despite the availability of experimental CCS values, theoretical CCS predictions offer an alternative approach to enhance lipid identification confidence by reducing false positives [47, 56]. Computational modeling, which entails constructing theoretical molecular structures and analyzing their spatial conformations through detailed computational simulations, has been used to predict CCS values. Commonly used methods include molecular dynamics, quantum chemical calculations using density functional theory, and molecular mechanics [56]. After optimization of molecular geometries, theoretical CCS values are derived using computational algorithms. However, simulations are time-consuming for large-scale molecules. Some studies have been devoted to improving calculation throughput. For example, a quantum chemistry-based theoretical calculation, the in silico chemical library engine (ISiCLE), employs MOBCAL for trajectory-based mobility calculations to predict molecular CCS values [57]. Moreover, the accuracy of computational modeling can be limited when applied to structurally complex and flexible lipids, such as fatty acids with numerous rotatable bonds and multiple intramolecular London dispersion interactions. Up to date, relatively few theoretical CCS values for lipids have been reported using computational modeling. Combining computational predictions with experimental data is increasingly utilized to improve predictive accuracy [43]. Recently, Keng et al. [58] developed a CCS knowledge-based approach for conformational sampling to improve accuracy, achieving average CCS prediction errors of approximately 2% for FAs and derivatives.

To overcome the limitations of computational modeling, machine learning has emerged as a powerful tool for prediction of lipid CCS values on a large scale. These machine learning approaches overcome the limitations of computational modeling methods by providing computationally efficient predictions with smaller errors. The workflow of machine-learning-based CCS prediction involves four key components: a training dataset containing experimentally measured CCS values, molecular descriptors (MDs), prediction algorithms (such as Support Vector Regression [SVR] and Artificial Neural Networks [ANN]), and validation datasets for external evaluation [12]. Molecular descriptors serve as critical inputs for machine-learning models, effectively capturing the physicochemical characteristics of lipids. Proper selection and optimization of these descriptors are crucial to avoid over-fitting and to enhance predictive accuracy. For example, careful reduction and selection of descriptors from hundreds to fewer relevant ones have significantly improved predictive outcomes. Zhu and coworkers utilized optimized 45 MDs and SVR models to predict the CCS values of 15,646 lipids across GP, SP, and GL [44]. The databases have been incorporated into accessible web-based prediction tools such as LipidCCS Predictor and AllCCS, broadening the availability of CCS predictions and allowing researchers to seamlessly integrate these valuable resources into their analytical pipelines [44, 59]. They also selected 12 MDs to predict the CCS values of 2068 sterol lipids derivatized with picolinic acid [49]. McLean and colleagues presented a large, unified CCS compendium to predict CCS values of compounds, including 810 lipid species, using nonlinear regression modeling according to the relationship between CCS and *m*/z [36]. Fernández and co-workers also reported a highly accurate machine learning algorithm (CCSP 2.0) in an open-source Jupyter Notebook format to predict CCS values based on linear support vector regression models [60].

However, current machine learning-based methods still face challenges, lacking molecular representations to efficiently represent the chemical properties that accurately predict molecular CCS values [61]. Two-dimensional (2D) MDs derived from simplified molecular-input line entry system (SMILES) do not fully capture the comprehensive structural features of lipids. Many research efforts have been devoted to optimizing molecular representation and machine learning methods to improve accuracy. For instance, many prediction models are limited in predicting the CCS values of isomeric lipids due to the lack of isomeric lipid standards in training datasets, limited mobility resolution, and insufficient molecular descriptors directly related to isomeric lipid structures. Using MDs closely related to the subtle structural differences of lipid isomers, Wang et al. [62] describe a least absolute shrinkage and selection operator (LASSO)-based approach for predicting the CCS values of lipids. This approach was demonstrated to allow differentiation of *cis/trans* and *sn*-positional isomers. However, the number of CCS values for lipid species produced based on lipid calibrants was quite limited in their prediction model. Li and co-workers selected 37 MDs and used an SVR-based model to construct a predicted CCS library comprised of 2500 GPs at *sn*-position isomer resolved level [52]. Another CCS database, CCSbase, contains nearly 7700 entries, covering a diverse chemical space and identifying structural characteristics, represented by molecular quantum numbers (MQNs), that contribute to the variance in mass-CCS space [45]. CCS prediction models were developed by decomposing chemical structural diversity through unsupervised clustering based on structural features, which may introduce a risk of misclassification. On the other hand, deep learning methods that use SMILES or molecular graphs as inputs and learn multilevel representations from chemical datasets to predict molecular properties have also been used for CCS prediction. For instance, DeepCCS predicted molecule CCS values from SMILES with deep neural networks (DNN) [63]. SigmaCCS integrated the advantages of the graph neural network (GNN) and the molecular graph, including 3D information for CCS prediction. It achieved a coefficient of determination of 0.9945 and a median relative error of 1.1751% on the test set [56]. To achieve more comprehensive molecular representations, AllCCS2 integrated MS, MD, and graph features, thereby establishing a universal method for predicting CCS values of small molecules. This approach reduced the median relative errors to 0.31%, 0.72%, and 1.64% for the training, validation, and testing sets, respectively [61].

Moving forward, the standardization of CCS determination and prediction methodologies will further improve the reliability of CCS values, making them more applicable for lipid identification. By integrating experimental data, optimizing molecular representations, incorporating advanced machine-learning strategies, and developing comprehensive CCS libraries, researchers can enhance the confidence of lipid identification and expand the role of IMS in lipidomics. Standardizing CCS libraries will not only improve accuracy but also facilitate broader applications in lipidomics, ultimately contributing to biomarker discovery and disease research.

## 4 | Multi-Dimensional Lipidomics and Spatial Mapping

### 4.1 | Integrating CCS as an Additional Dimension for High-throughput Lipidomics

CCS values serve as a crucial orthogonal parameter, complementing *m/z*, RTs, and tandem mass spectra (MS/MS), and have revolutionized lipid identification. The combination of experimental and predicted CCS databases has played a key role in reducing false-positive identifications and improving structural annotation confidence. In recent years, continuous studies have demonstrated that incorporating CCS into lipidomics workflow facilitated structural characterization, enhanced separation of isomeric lipids, reduced false positive annotation, and broadened the lipidome coverage, enabling comprehensive lipid identification in biological analysis.

For instance, combined CCS matches with *m/z*, 89.5% false positive identifications were correctly filtered for PC standard mixture [44]. In complex biological samples, including human plasma, human 293T cell, mouse brain, and heart tissues, an average of 12.5 potential candidates was reduced after the addition of the CCS value match of 1% tolerance [44]. In the IM-MS structural database comprising 456 mass-resolved CCS values of sphingolipid and glycerophospholipid species, CCS information enables more confident identification of isomeric lipid species and isobars within narrow mass windows [64]. Studies also demonstrated that the addition of CCS match improved the annotation accuracy for metabolites and lipids in untargeted analysis [59]. Approximately 75% of the annotated candidates were filtered with the addition of CCS match to *m/z* match. The addition of CCS match into the multi-dimensional (*m/z* + MS/MS) match also improved the rank of correct candidates [59]. Using the annotation of 6-hydroxycoumarin as an example, the inclusion of CCS matching reduced the number of potential candidates from 956 to 181 [59, 65].

**FIGURE 4 |:**
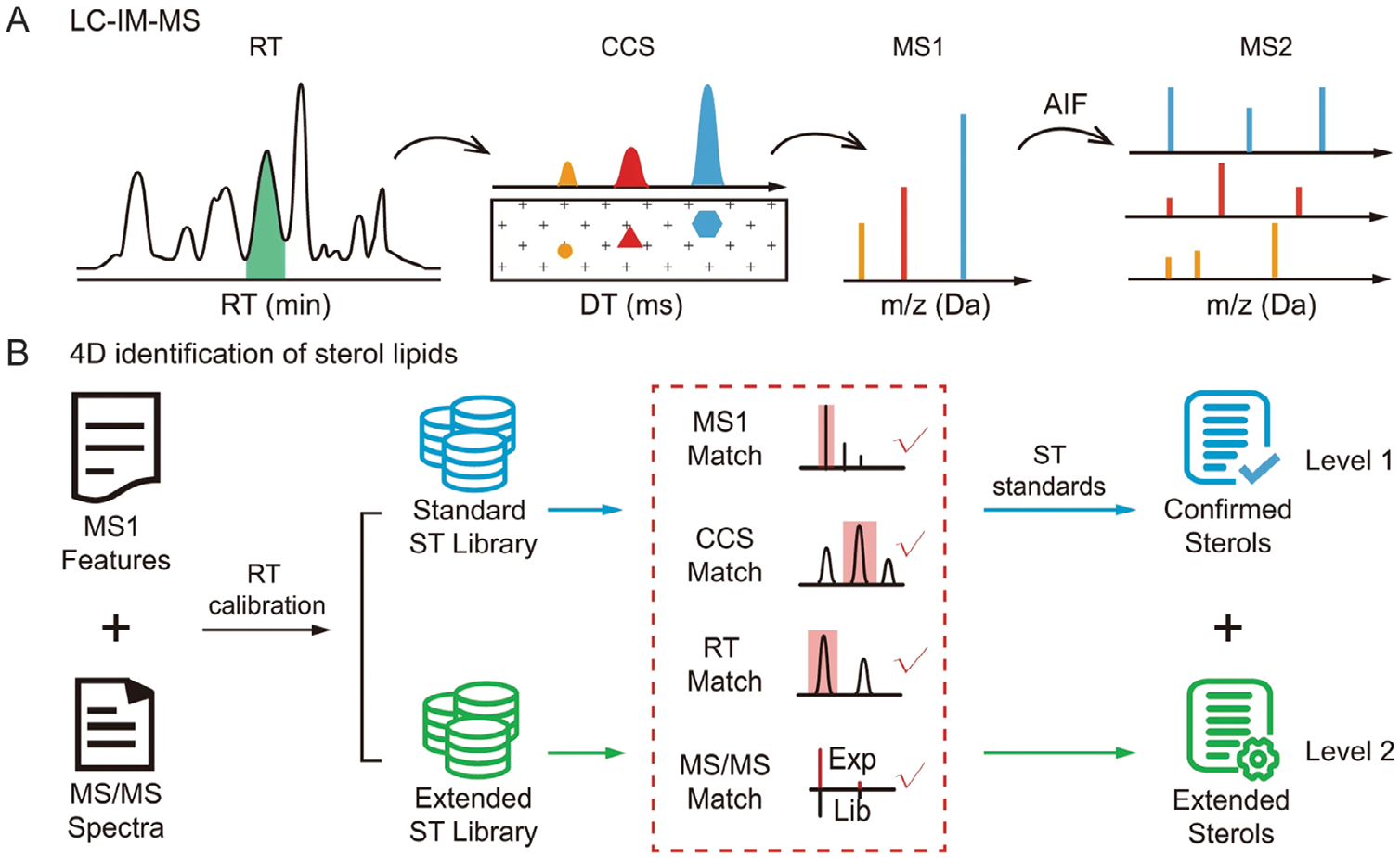
(A) Four-dimensional sterol lipidomics dataset comprising retention time (RT), collision cross-section (CCS), MS1, and MS2 spectral data acquired through liquid chromatography-ion mobility-mass spectrometry (LC-IM-MS). (B) Four-dimensional sterol library enabling comprehensive identification and confirmation of sterol lipid species. Adapted from Li et al. [49] with permission.

Zhu and colleagues established a comprehensive sterol lipid library through the systematic integration of 4D analytical data, including RT, *m/z*, MS/MS fragmentation patterns, and CCS values (Figure 4) [49]. Augmented by machine learning algorithms, this approach enabled the construction of a high-confidence database encompassing over 2000 sterol lipids. Leveraging this advanced library, the researchers mapped the spatial distribution of sterol lipids across ten distinct functional regions of the mouse brain and revealed approximately 200 sterol lipids, with concentrations spanning eight orders of magnitude [49]. Similarly, Baker and co-workers developed a comprehensive 4D human plasma lipid library, which comprises over 500 unique lipids and incorporates adapted Skyline functionalities, such as indexed retention time (iRT) prediction and IMS drift time filtering for enhanced confidence. The established workflow was applied to plasma and bronchoalveolar lavage fluid (BALF) samples from patients with varying degrees of smoke inhalation injury to identify lipid biomarkers with diagnostic and prognostic value. A total of 25 lipids showed statistically significant differences between patient outcomes, including 19 lipids in plasma and 6 in BALF [50].

Leveraging HRdm mode in Agilent 6560 DTIMS, Li and co-workers constructed a comprehensive experimental 4D GP database of 498 GPs identified from the mouse brain and an in-depth extended 4D library of 2500 GPs predicted by machine learning, enabling automated profiling of GPs with detailed acyl chain *sn*-position assignment. With both the experimental database and the extended library, a significantly high number (>540) of GP species with *sn*-position information were identified and quantified from three functional regions of the mouse brain, revealing the spatial and temporal GP alterations in the brains of a mouse model for Alzheimer’s disease [52]. Some recent studies also applied DTIMS and TIMS-based 4D lipidomics strategies to improve the coverage and accuracy [26, 66]. In addition, the 4D lipidome strategy for confident lipid annotation can expedite clinical profiling. Lerner et al. [67] showcased the applicability of the 4D TIMS lipidomics for reproducible and comprehensive pheno-mapping in clinical samples by a pilot study of intra-individual and multidien lipidome in blood matrices: plasma, serum, direct blood, fingertip dried blood spot (DBS), and venous DBS. A total of 370 lipids in reference plasma samples and 364 lipids in serum samples were confidently annotated. Moreover, the study showed that LC and TIMS separation could prospectively aid in annotation coverage of isomeric species for *cis/trans* GP isomers [67].

Table 2 summarizes the key features distinguishing shotgun lipidomics, reversed-phase liquid chromatography (RPLC), and IMS-enabled RPLC-MS strategies. The integration of multi-dimensional information, including CCS measurements, chromatographic separation, MS, and MS/MS fragmentation, has significantly enhanced lipidomics, enabling more accurate and comprehensive lipid profiling. Continued advancements in analytical techniques, database development, and computational tools will further propel the field, offering deeper insights into lipid biology and its implications for health and disease.

### 4.2 | Advancing Spatial Lipidomics With IMS-Based Mass Spectrometry Imaging

Cellular heterogeneity significantly impacts the microenvironment of biological tissues and provides valuable insights into inter- and intra-cellular communications. Understanding the interplay and behaviors of specific cell populations can enhance our knowledge of tissue homeostasis. To achieve this, spatially resolved techniques with micrometer resolution are often necessary for profiling across the tissue [68, 69]. Mass spectrometry imaging (MSI) is an emerging spatially resolved technique that operates in an untargeted manner based on mass-to-charge ratios. It offers comprehensive characterization of molecular features and visualization of molecular spatial distributions [70]. The applications of MSI to multi-omics studies have been extensively reported, with lipidomics being a critical area of study in cellular biology, as cells and organelles are the fundamental units of life [71].

Conventional MSI for lipidomics typically involves a three-step molecular identification process: (1) MS1-level accurate mass matching (AMM) against a database with a stringent *m/z* error tolerance, primarily constrained by the instrumentation; (2) parallel tissue extraction followed by LC-MS/MS identification matching; (3) direct in situ MS/MS structural elucidation. Although in situ MS/MS structural elucidation provides the most accurate structures among the three methods, it is often hindered by limited sample amounts and poor ion utilization in a single pixel [72]. Furthermore, the complexity of lipid mixtures in a typical biological tissue section can lead to isobaric and isomeric interferences, as numerous species may share similar or identical mass-to-charge ratios [73]. This *m/z* overlap complicates the accurate identification and quantification of individual lipid species. Among these two challenges, the isobaric interferences can further be resolved with the advanced instrumentation using higher mass resolution mass analyzers, such as Fourier transform ion cyclotron resonance (FT-ICR) and orbitrap systems, which often require extra expenses and longer acquisition time limited by the instrument cycle time for ion utilization. On the other hand, the isomeric interferences such as double bond (C═C) locations and geometry in phospholipids cannot be resolved simply by mass resolution improvement of instrumentation and techniques because of the identical mass-to-charge ratio at the MS1 level [74–76]. The current solutions to pinpoint the structural differences often rely on chemical derivatization and various ion dissociation methods such as ultraviolet photodissociation (UVPD) and ozone-induced dissociation (OzID), which require hardware modification and relevant expertise [77–79]. Additionally, the ionization efficiencies of different lipids can vary, potentially resulting in biased representations of lipid abundances. These challenges underscore the necessity for enhanced analytical techniques for MSI that can provide additional separation dimensions and improve molecular specificity in lipidomics studies.

**FIGURE 5 |:**
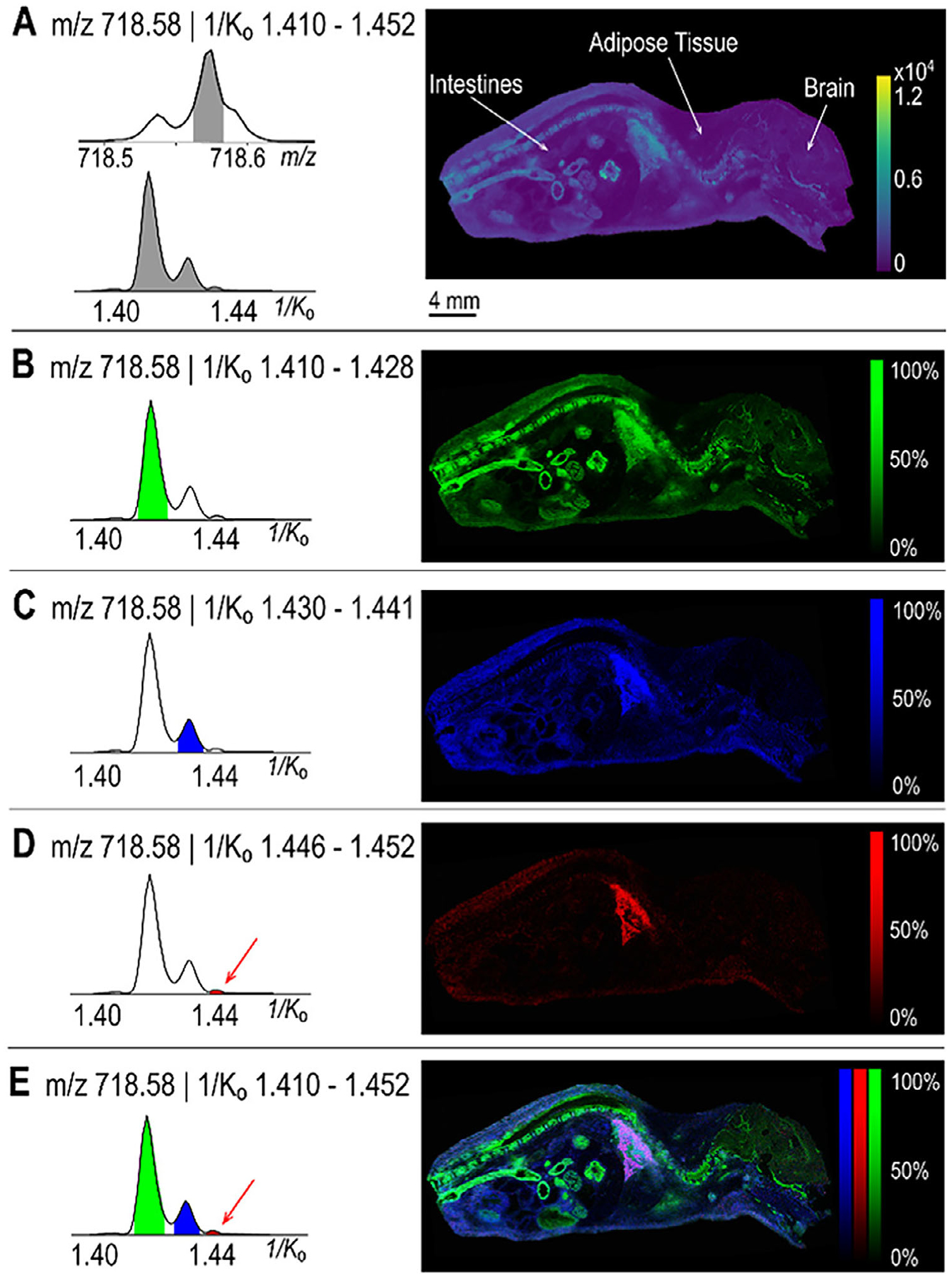
Ion mobility spectrometry facilitates the separation of lipid isomers in whole-body mouse pup tissues. A composite image displaying all three peaks in the extracted ion mobilogram (1/K_0_ 1.410–1.452) of *m/z* 718.58 is shown in panel (A). Individual panels highlight ion images corresponding to three distinct mobility ranges: 1/K_0_ 1.410–1.428 (B), 1.430–1.441 (C), and 1.446–1.452 (D), with an overlay of all three mobility peaks (left) and ion images (right) presented in (E). Adapted from Djambazova et al. [88] with permission.

Owing to the nature of IM-MS relying on the mobility-based separation, this time-series signal acquisition is thereby suitable for the subsequent TOF ion detection, which is the prevalent mass analyzer for MSI experiments [80–82]. By distinguishing ions that may have identical or minimal differences in *m/z* values but differ in their mobility drift times, IM-MS enhances the separation of complex mixtures, facilitating more precise identification of lipid species [83–85]. This additional separation of ions enables the possibility for more accurately characterizing the isobaric and isomeric structures, as isobars and isomers can be isolated by both precursor masses and their gas-phase molecular mobilities for the subsequent collision-induced dissociation (CID), respectively [86, 87]. Beyond that, IM-MS ensures the annotation confidence when matching the ion signals with analytical standards and integrating isotopologue strategies into the workflow [73, 64]. Recent advancements in instrumentation have further opened the door for IM-MS applications to MSI. IM-MSI can reveal distributional differences for isobars and isomers after mobility-based separation, providing a more holistic view of localization for previously indistinguishable molecules [88–90]. For instance, the IM-MSI was proposed to resolve the phospholipid isomers in whole-body mouse pup tissues (Figure 5), where the TIMS enabled the separation with resolution >200 within 50–500 ms of putative identifications for [CerP (t40:1) + H]^+^, [PC (O-32:1) + H]^+^, and [PC (P-21:0) + H]^+^ by their distinct 1/K_0_ values [88, 91, 92]. The three species resolved in the extracted ion mobilogram (EIM) exhibited different distribution patterns, which would be misinterpreted by mass-only MSI due to isomeric overlap. Gangliosides, another class of essential acidic glycosphingolipids in the immune system, were also investigated with IM-MSI in a *Staphylococcus aureus*-infected mouse kidney section. The GM1 structural isomers (e.g., GM1b Neu5Ac(t34:1), GM1b Neu5Gc(d34:1), GM1a Neu5Gc(t34:1), and GM1a Neu5Gc(d34:1)) were found separated with distinct 1/K_0_ values and showed varied distributions that help to elucidate the functions of different isomers at the infection site [89, 90]. GD1a/b isomers, as another example of disialoganglioside isomers, have been found and investigated in murine nervous tissue and rat spinal cord. MALDI-IM-MSI was successfully used to characterize both GD1a/b(36:1) and GD1a/b(38:1) isomeric species, enhancing our understanding of Alzheimer’s disease in ways that cannot be achieved with IHC or other types of optical microscopy techniques [89].

Recent technological advancements have significantly enhanced the capabilities of IM-MS in spatial lipidomics [93, 94]. The implementation of advanced ion mobility techniques, such as cyclic ion mobility spectrometry, has enhanced the resolution and sensitivity of lipid analyses, providing IM resolution of 250 or greater to separate most lipid isomers [95]. With the cyclic ion mobility separation, the PS (P-40:6) was observed to be differentiated from its isobar after six cIM passes and showed non-isobar overlapped ion images for the mouse brain tissue section. Additionally, the advanced instrumentation reduces acquisition time, facilitating high-throughput MS1 collection with parallel reaction monitoring and parallel accumulation serial fragmentation (prm--PASEF) for molecular identification [65, 96]. Recent studies have highlighted the significant applications of prm-PASEF in advancing lipidomic identification using MSI. Li et al. successfully integrated imaging parallel reaction monitoring (iprm)-PASEF with MALDI MSI, enabling multiplexed MS/MS acquisition and enhanced analyte identification directly from tissue sections [96]. This approach addressed the limitations of conventional MALDI-MSI by improving precursor selection efficiency and reducing spectral complexity. Similarly, Heuckeroth et al. [65] introduced a dataset-dependent acquisition strategy for on-tissue MALDI-TIMS-MS2 imaging, which systematically scheduled fragmentation events based on spatial distribution and spectral purity. This method enabled comprehensive lipid annotation within complex biological samples, particularly in neurological tissues, where lipid distributions are crucial for understanding neurodegenerative processes. Meanwhile, Qian et al. [97] developed a mobility-modulated sequential dissociation (MMSD) strategy, which leveraged ion mobility-assisted data-independent acquisition to achieve high-throughput lipid MS/MS imaging. Their findings highlighted the capability of MMSD to resolve structural lipid isomers and map their spatial distribution within tissues, revealing key lipidomic alterations in hepatocellular carcinoma. Collectively, these studies underscore how prm-PASEF and related advancements in ion mobility have significantly enhanced lipidomic MSI, enabling deeper insights into lipid heterogeneity and disease pathology [98].

### 4.3 | Quality Control and Quantitative Considerations in IMS-enabled Lipidomics

Despite advances in leveraging IMS and CCS measurements to expand lipidome coverage, rigorous quality control of lipid feature assignment remains critical. Recent debates underscore the importance of experimental validation in lipid identification. For instance, in the study by Vasilopoulou et al. [25], which employed TIMS, PASEF, and nano-LC for comprehensive lipidome profiling, it was suggested that at least 510 out of 1108 reported lipid features may require additional experimental confirmation [99]. Over-reliance on software-assisted annotation without adequate orthogonal evidence can lead to substantial false identifications.

To enhance the reliability and biological relevance of lipidomics data, lipid identifications should be validated against rational criteria—such as consistent retention times, accurate mass, and diagnostic MS/MS features (including adduct types and characteristic fragments) [99]. While this may reduce the number of reported features, it ensures data integrity and downstream interpretability.

In parallel, several tools have been developed to improve CCS-based quality control in IMS-lipidomics workflows. One notable example is MobiLipid [100], an open-source tool designed for the internal standardization and quality control of CCS values. MobiLipid utilizes a curated drift tube IMS library of 377 uniformly ^13^C-labeled lipids to automatically monitor and correct CCS measurement biases. Its effectiveness was demonstrated in a TIMS-MS analysis of yeast extracts spiked with ^13^C-labeled lipids, where it significantly improved the accuracy and reliability of lipid annotations by reducing the mean absolute CCS bias to 0.78% in positive mode and 0.33% in negative mode.

In addition to quality control, the quantitative capabilities of IMS should also be considered. When incorporating IMS devices into different mass spectrometry platforms, the dynamic range can vary depending on the specific instrumentation. Among the commonly used IMS techniques—DTIMS, TWIMS, and TIMS—integration with Time-of-Flight (TOF) mass analyzers is most prevalent. Notably, previous studies have shown that adding an IMS device to a TOF analyzer does not compromise the linear dynamic range [101]. In fact, IMS often enhances the effective dynamic range for complex biological samples. By separating high-abundance interferences from low-abundance analytes in the mobility dimension prior to mass analysis, IMS reduces space-charge effects and mitigates signal suppression. This benefit is particularly well-demonstrated in DMS [102], although further investigation is needed to confirm similar advantages across other IMS platforms.

Regarding matrix effects in lipidomic quantification, IMS proves to be a powerful approach for resolving analytes from co-eluting or isobaric interferences that are challenging to separate using LC-MS alone or in shotgun lipidomics workflows [26]. This separation capability significantly improves quantification accuracy.

In addition to the commonly used label-free quantitation, IMS-enabled lipidomics also supports stable isotope-based quantification strategies. For instance, Xu et al. [53] developed an amine-reactive isotopic *N*,*N*-dimethyl leucine (iDiLeu) reagent to label *sn*-positional aminophospholipid (APL) isomers. This isobaric tag enhances the overall analytical sensitivity and throughput. Specifically, the 5-plex iDiLeu labeling strategy enables the construction of an internal 4-point calibration curve, facilitating absolute quantification of APL *sn*-isomers within a single analytical run.

## 5 | Conclusion

Recent advancements in IM-MS have significantly enhanced the precision, sensitivity, and throughput of MS-based lipidomics. As instrumentation continues to evolve, the resolution for distinguishing lipid isomers is expected to reach unprecedented levels, establishing IM-MS as an indispensable orthogonal analytical dimension that complements traditional mass spectral data. Concurrently, the growing volume of experimentally derived CCS measurements is facilitating the expansion of lipid-specific CCS databases. This progress, augmented by computational modeling and machine learning–driven predictive tools, promises to accelerate the annotation of lipid species and resolve previously ambiguous isomer assignments. As these technologies mature, the integration of IM-MS into lipidomic workflows is anticipated to yield transformative molecular insights. Such advances will not only drive the discovery of lipid-based biomarkers but also deepen our understanding of lipid metabolism, signaling pathways, and their roles in health and disease.

## Figures and Tables

**TABLE 1 |:** Publicly available experimental CCS databases for lipids.

	Lipid MAPS	CCS base	METLIN-CCS lipid database
Unique lipid ion adducts	1235	5303	2413
Lipid class coverage	6	Over 40	Over 20
CCS data source	DTIMS	DTIMS, TWIMS	DTIMS
Website	https://www.lipidmaps.org/	https://ccsbase.net/	https://metlin.scripps.edu/

**TABLE 2 |:** Comparative analysis of lipidomics strategies.

	Shotgun lipidomics (direct infusion)	Reversed-phase LC-MS (RPLC-MS)	RPLC with IMS
Isomer resolution	**Very Low**. Unable to separate most isomers and isobars.	**Moderate to High**. Separates species by polarity/hydrophobicity.	**High**. Separates ions by size, shape, and charge (CCS). Orthogonal to LC and MS.
Throughput	**Very High**. Analysis time is on the order of seconds to a few minutes per sample.	**Low to Moderate**. Analysis time is typically 15–60 min, depending on chromatographic gradient.	**Low to Moderate**. Analysis time is dictated by the LC gradient, similar to standard RPLC-MS.
Lipidome coverage	**Moderate**. Limited by significant ion suppression effects, which mask low-abundance species.	**High**. Chromatographic separation reduces ion suppression, improving detection of a wider range of lipids.	**High to Very High**. Further reduces chemical noise and separates lipids from matrix, improving signal-to-noise and enhancing coverage.
Quantitative capability	**Moderate**. Prone to inaccuracies due to unresolved isobars and strong matrix effects.	**High**. Reduced matrix effects and separation of isobars lead to more accurate and reliable quantification.	**High**. Provides cleaner MS/MS spectra by separating co-eluting precursors, leading to more specific and accurate quantification.
Primary limitation	Lack of separation leads to high spectral complexity and isomeric ambiguity.	Long analysis times are a bottleneck for large-scale studies. Incomplete isomer resolution.	Requires robust calibration and validation workflows to ensure accuracy of CCS values and avoid false identifications.
